# Unexpected Long-Term Protection of Adult Offspring Born to High-Fat Fed Dams against Obesity Induced by a Sucrose-Rich Diet

**DOI:** 10.1371/journal.pone.0018043

**Published:** 2011-03-25

**Authors:** Odile Couvreur, Jacqueline Ferezou, Daniel Gripois, Colette Serougne, Delphine Crépin, Alain Aubourg, Arieh Gertler, Claire-Marie Vacher, Mohammed Taouis

**Affiliations:** 1 Neuroendocrinologie Moléculaire de la Prise Alimentaire, University of Paris-Sud, UMR 8195, Orsay, France; 2 Neuroendocrinologie Moléculaire de la Prise Alimentaire, CNRS, Centre de Neurosciences Paris-Sud UMR8195, Orsay, France; 3 The Institute of Biochemistry, Food Science, and Nutrition, Faculty of Agricultural, Food and Environmental Quality Sciences, The Hebrew University of Jerusalem, Rehovot, Israel; Chinese University of Hong Kong, Hong Kong

## Abstract

**Background:**

Metabolic and endocrine environment during early life is crucial for metabolic imprinting. When dams were fed a high fat diet (HF diet), rat offspring developed hypothalamic leptin resistance with lean phenotype when weaned on a normal diet. Interestingly, when grown on the HF diet, they appeared to be protected against the effects of HF diet as compared to offspring of normally fed dams. The mechanisms involved in the protective effect of maternal HF diet are unclear.

**Methodology/Principal Findings:**

We thus investigated the impact of maternal high fat diet on offspring subjected to normal or high palatable diet (P diet) on metabolic and endocrine parameters. We compared offspring born to dams fed P or HF diet. Offspring born to dams fed control or P diet, when fed P diet exhibited a higher body weight, altered hypothalamic leptin sensitivity and metabolic parameters suggesting that maternal P diet has no protective effect on offspring. Whereas, maternal HF diet reduces body weight gain and circulating triglycerides, and ameliorates corpulence index of offspring, even when subjected to P diet. Interestingly, this protective effect is differently expressed in male and female offspring. Male offspring exhibited higher energy expenditure as mirrored by increased hypothalamic UCP-2 and liver AdipoR1/R2 expression, and a profound change in the arcuate nucleus astrocytic organization. In female offspring, the most striking impact of maternal HF diet is the reduced hypothalamic expression of NPY and POMC.

**Conclusions/Significance:**

HF diet given during gestation and lactation protects, at least partially, offspring from excessive weight gain through several mechanisms depending upon gender including changes in arcuate nucleus astrocytic organization and increased hypothalamic UCP-2 and liver AdipoR1/2 expression in males and reduced hypothalamic expression of NPY and POMC in females. Taken together our results reveal new mechanisms involved in the protective effect of maternal HF diet.

## Introduction

Obesity has been considered to result from both a genetic prevalence and inadequate nutrition due to lifestyle, and more recently epidemiological evidence raised the notion of a developmental origin of this pathology and associated diseases [1]. According to the «thrifty phenotype» hypothesis [2], a poor fetal nutrition leads to programming of an adult phenotype that is adapted to poor nutrition, but a mismatch between predicted and postnatal environment then promotes a persistent dysregulation of the body weight control [3]. Thus, low-birth-weight babies due to adverse foetal conditions often display an increased susceptibility to develop a metabolic syndrome when submitted to plentiful conditions later in life [4], [5]. Such a developmental programming, reproduced in animal models by maternal undernutrition [6], is in part attributed to a relative lack of leptin during crucial time windows in the developmental neuronal plasticity, since a normal adult phenotype may be restaured after treatment of either pregnant dams [7] or suckling pups [8], [9] by exogenous leptin. This pleiotropic adipocyte-derived cytokine acts as an essential neurotrophic factor along the development of the hypothalamic circuits regulating metabolic homeostasis [10]. Later in life, leptin through its binding to specific ObRb receptors (long isoform of leptin receptor) especially abundant in the arcuate nucleus triggers the concerted signalling pathways leading to reduce appetite and increase energy expenditure [11]. Moreover, the growing proportion of women that are overweight before and during pregnancy and lactation [12] raised the question of the impact of leptin excess during critical perinatal periods on the risk of becoming obese in adulthood. Indeed this risk has been shown to be increased in pups issued from dams treated with leptin before weaning [13] or overfed by suckling in small litters [14]. While maternal high-fat diets have been often reported to program obesity in offspring [15]–[19], discrepant results obtained in rodents may be related to the choice of the strain, the litter size and the composition of the inappropriate diet promoting maternal obesity, mimicking the features of hypercaloric foods available in modern societies [20], [21].

In a previous study carried in Wistar rats [22], pups reared in large litters and born to dams fed a high-fat (HF) diet, from before conception and throughout gestation and lactation, displayed a lower weaning body weight as compared to their counterparts born to control dams. Their growth retardation was related to the abnormal fall in body weight observed in lactating dams. After 6 weeks feeding a control diet from weaning, these pups were characterized by a defect of leptin signaling in hypothalamus despite a lean phenotype and normal leptin, insulin, glucose and lipid plasma levels [22]. Interestingly, when the same HF diet was provided for 6 weeks after weaning, only males issued from normally fed dams become overtly obese while those born to HF dams were protected against the obesogenic effect of the HF diet, despite the same defective hypothalamic leptin signaling. Their «spendthrift» phenotype suggested a persistent modification of the energy control, in agreement with a predictive adaptive response [23] to the inappropriate HF diet [24]–[26].

**Figure 1:**
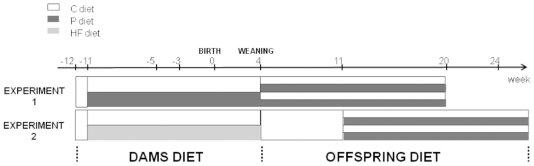
Model depecting the experimental protocol for experiment 1 and experiment 2. C, P and HF diets correspond to Chow, High palatable diet and High Fat diet, respectively.

In the present paper, a highly palatable (P) diet was used (experiment 1, Figure 1) to induce maternal obesity [27], then the adult phenotype of male offspring was compared to that of control rats born to chow-fed dams. When pups issued from obese dams were assigned to the chow diet at weaning, they displayed inherited defective leptin signaling in hypothalamus, which persisted until age of 6 months despite normal body weight evolution. However, pups born to obese P dams and weaned on the same P diet were not protected against diet induced obesity. Clearly the two inappropriate HF and P diets have distinct impacts on both dams and pups, likely in relation with their different palatability or composition. Both diets (HF and P) were then used (experiment 2, Figure 1) in order to study whether adult pups born to HF dams and weaned on a chow diet, will be more or less susceptible than control rats to develop obesity when switched to the obesogenic P diet, a question that remained unresolved in our previous study [22]. Thus, male and female offspring born to HF or control dams were assigned to the control diet for 7 post-weaning weeks, and then switched to the P diet for 3 additional months. Our results clearly showed that offspring from both genders born to HF dams were protected from the obesogenic effect of P diet as their body weight gain was lower as compared to offspring born to chow dams. In addition, the potential protective effect of maternal HF diet involves most likely different gender-dependent mechanisms.

## Results

### Experiment 1

#### Females and pups until weaning

**Figure 2:**
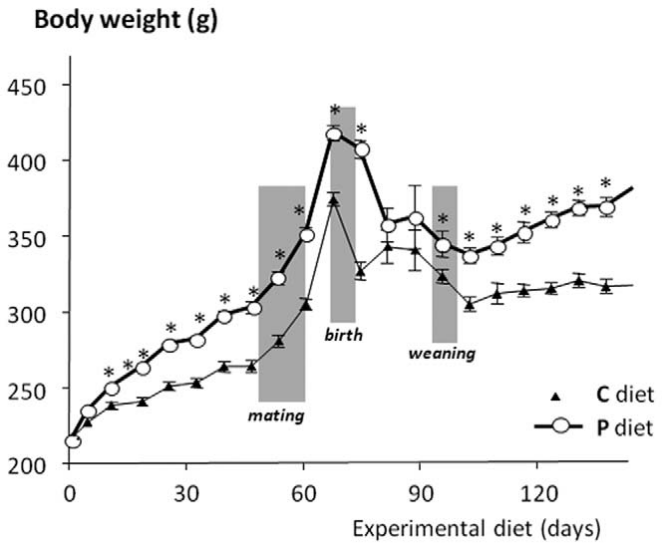
Evolution of the body weight of dams fed the control (C) diet (n = 16) or highly palatable diet (P) (n = 16) for 6 wk before mating, throughout gestation and lactation (28 days) and until the postweaning period (*p<0.05).

According to the cumulative food intake measured during 4 weeks before mating and the energy density of each dry diet (Table 1), the mean daily amount of food ingested per rat (measured every two days on 4 cages of 4 rats in each group) was higher for the P diet (25.1±0.4 g as wet P diet or 16.6±0.3 g as dry P diet) than for the C diet (15.18±0.18 g, p<0.0001), then providing more energy (65.2±1 and 56.2±0.7 kcal, p<0.0001, respectively). The number of females which became pregnant (12 out of 16 animals in each group) and the initial size of the litters (11.7±0.8 and 11.7±0.9 pups from P and C dams, respectively) were not influenced by the maternal diet, but the male/female ratio (0.79 for 140 pups born to P dams versus 1.16 for 141 pups born to C dams) was inversed. No difference appeared in the mean birthweight and after the litter size was equilibrated to 11–12 pups at birth, the evolution of the mean litter weight was identical regardless the maternal diet until weaning (results not shown). As shown in Figure 2, P dams became overtly obese compared to C dams (n = 12 per group) and their overweight (about 15% before mating) was maintained after 120 days of experimental diet, i.e 20 days after weaning. At this time, fasting plasma level of leptin was clearly higher in P dams (6.5±1.3 ng/mL, n = 6) than in control dams (1.9±0.5 ng/mL, n = 7, p<0.005).

**Table 1:** Energy content of the commercial chow diet (C) and the semi-purified highly palatable (P) and high-fat (H) diets.

Energy (kcal %) derived from	C	P	H
Carbohydrates	66.2	70	22.6
Proteins	22.7	14.6	12.9
Lipids	11.1	15.4	64.5
Energy content (kcal/100 g of dry diet)	370.1	392.6	571.9

#### Offspring after weaning

**Figure 3:**
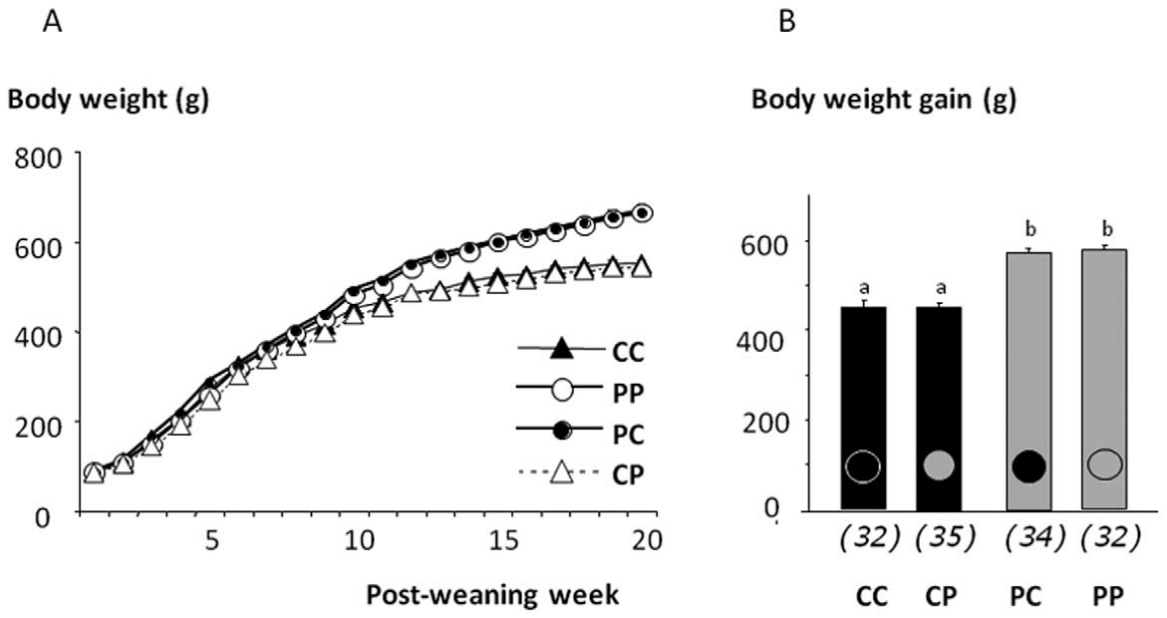
Impact of post weaning diet on the body weight of male pups born to dams fed the control diet (C) or highly palatable diet (P). At weaning four groups of male pups (CC, n = 32; CP, n = 35; PC, n = 34; PP, n = 32) were formed and named according to the post weaning diet (C or P, first letter) and to maternal diet (C or P, second letter) and their body weight and body weight gain were registred during 20 weeks after weaning. Body weight and final body weight gain are reported in panel 3A and 3B, respectively (*p<0.05).

Daily energy intakes and final physiological parameters measured in adult fasted rats are shown in Table 2. Mean daily energy intakes were calculated from daily food intakes measured twice a week from the 2^nd^ to the 6^th^ post-weaning week on 10 cages of 2 rats per group. The highest value was found in the PC group of rats fed the highly palatable P diet and born to normally fed dams. Their counterparts born to obese dams (PP group) ingested less energy for a similar weight gain, suggesting a better food efficacy of the P diet in this group. The maternal diet did not affect CC and CP rats, which both displayed a lean phenotype while their energy intake was close to that of obese PP rats. As observed in Figure 3, independently of maternal diet a striking effect of the post-weaning diet appeared on the body weight evolution and gain. The same conclusion was drawn by comparing plasma concentrations of triglycerides, leptin and insulin, all reported in Table 2. These parameters were higher in rats fed the P diet (PP and PC groups) than in rats fed the chow diet (CC and CP groups). The only differences concerned the plasma levels of leptin which were lower in CP rats than in control CC rats, and the plasma levels of insulin and HOMA index of PP rats which overpassed those of PC rats. In addition, the lowest plasma cholesterol value was found in PP rats, indicating a long-term impact of the maternal metabolic status on these parameters.

**Table 2:** Physiological parameters measured in fasted and fed male rats (age: 11 weeks) from 4 experimental groups named according to the post-weaning and maternal chow or palatable diet (C or P as 1^st^ and 2 nd letter, respectively).

Group	CC	CP	PC	PP
**Daily energy intake** (Kcal/day)	87.2±1.3^a^	83.2±4.5^a^	99.1±1.9^b^	88.4±2.5^a^
(n)	(16)	(16)	(16)	(16)
**Overnight Fasted**				
(n)	(20)	(20)	(20)	(20)
Liver weight (g)	12.0±0.3^a^	12.0±0.4^a^	14.3±0.3^b^	13.9±0.3^b^
%Body weight	2.32±0.05^bc^	2.39±0.05^c^	2.20±0.04^ab^	2.10±0.04^a^
Liver lipids (mg/g)	3.04±0.22	2.53±0.18	3.08±0.18	2.80±0.20
**Plasma**				
(n)	(20)	(20)	(20)	(20)
Glucose (g/L)	0.928±0.017	0.996±0.031	1.019±0.026	1.006±0.025
Insulin (ng/mL)	0.631±0.09^a^	0.761±0.071^a^	1.703±0.128^b^	2.328±0.224^c^
HOMA*	3.54±0.38^a^	4.71±0.55^a^	10.39±0.80^b^	14.07±1.33^c^
Leptin (ng/mL)	3.57±0.64^b^	2.64±0.57^a^	11.49±1.51^c^	14.69±4.64^c^
Triglycerides (g/L)	0.84±0.047^a^	0.742±0.049^a^	1.268±0.094^b^	1.481±0.112^b^
Cholesterol (g/L)	0.862±0.058^b^	0.813±0.036^b^	0.707±0.036^b^	0.654±0.025

*calculated according to Tumer et al. (1993),

**subgroup of rats injected with physiological saline (n = 10). Different superscript letters ^a,b,c^ denote significant differences at p<0.05 by ANOVA and the Fisher posthoc test.

#### Hypothalamic leptin sensitivity of offspring

**Figure 4:**
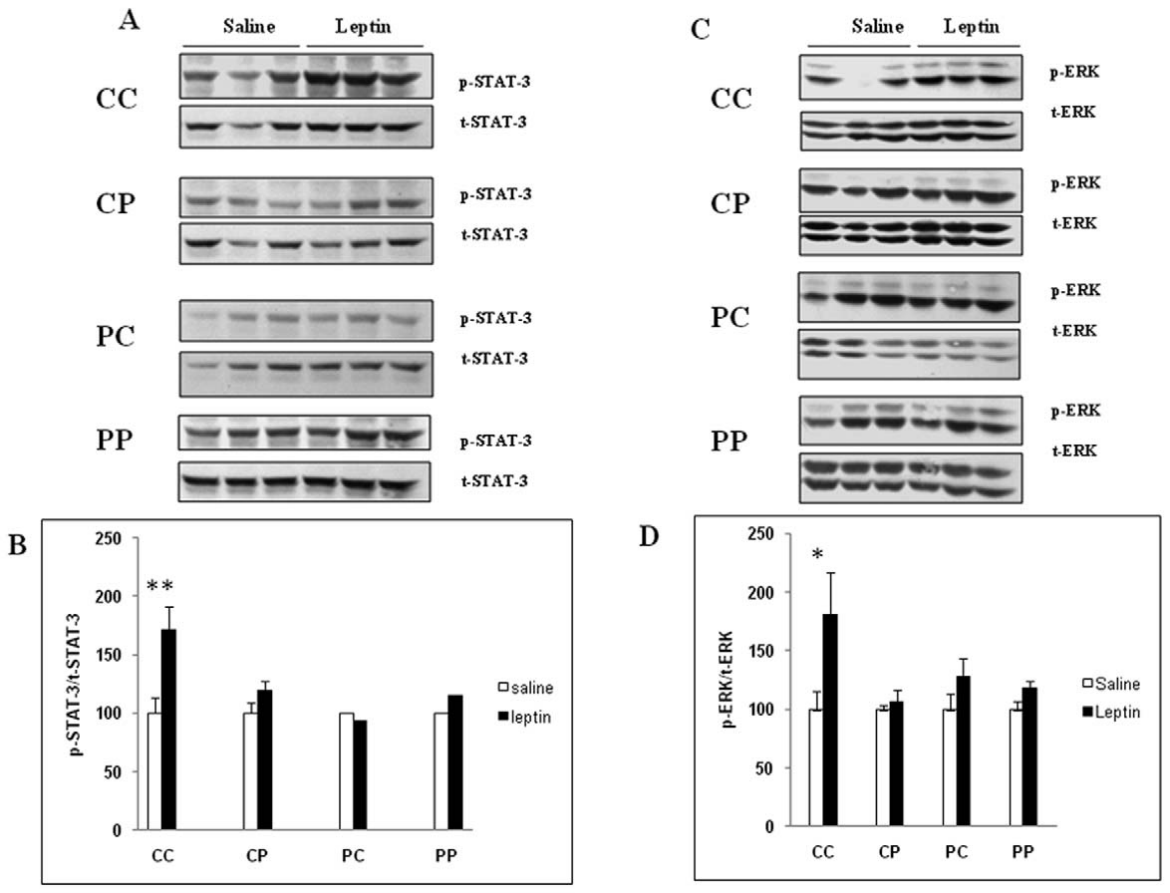
Phosphorylation of STAT-3 and ERK in the hypothalamus of male offspring born to dams fed the control diet (C) or highly palatable diet (P). At weaning four groups of male pups (CC, CP, PC, PP) were formed and named according to the post weaning diet (C or P, first letter) and to maternal diet (C or P, second letter). Each goup contained 20 rats was divided into two sub-groups that received by IP either saline (n = 10) or leptin (n = 10). In each group, the sensitivity toward leptin was assessed by a significant elevation of the mean p-STAT-3/t-STAT-3 and p-ERK/t-ERK ratio in leptin-injected compared to saline-injected rats. Panel A and C show representative western-blots for total and phosphorylated STAT-3 and ERK, respectrively. Panels B and D show the mean ratio band density of phosphorylated and total STAT-3 and ERK, respectively; (**p<0.005; * p<0.05).

The hypothalamic leptin sensitivity was assessed in each group by comparing the phoshorylation levels of STAT3 and ERK1/2 in response to a bolus of leptin. Briefly, 11 week-old rats from the four groups were starved overnight and divided in two groups that receive by IP saline or leptin, respectively. The phoshorylation levels of STAT-3 and ERK1/2 were measured by Western blot. Leptin IP bolus induced STAT-3 (fig. 4A and 4B) and ERK1/2 (fig. 4C and fig. 4D) phosphorylation only in CC groups, whereas the other groups displayed a clear hypothalamic leptin-resistance (Fig. 4).

### Experiment 2

#### Females and pups until weaning

**Figure 5:**
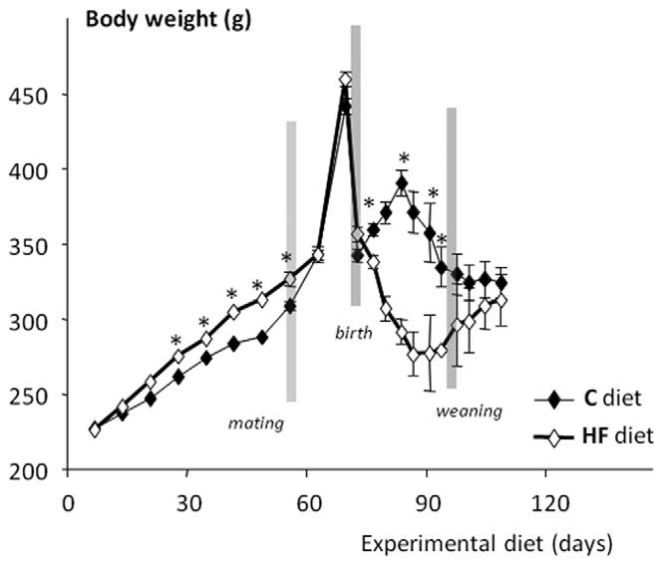
Evolution of the body weight of dams fed the control (C) diet (n = 28) or high-fat diet (HF) (n = 28) for 6 wk before mating, throughout gestation and lactation (28 days) and until the postweaning period (*p<0.05).

The experiment 2 was performed in order to study the impact of high fat diet (HF) during pregnancy and lactation on offspring when swithched to a high palatable diet (P) at the adulthood. The measurement of food intake twice a week before mating (14 cages of 2 rats per group) indicated that dams fed the high fat diet daily ingested less food than control dams fed the chow diet (12.6±0.2 vs 18.2±0.2 g/rat, p<0.0001), which however provided more energy (71.9±1.1 kcal/day vs 67.4±0.7 kcal/day, p<0.002) due to the high caloric density of the HF diet. After 7 weeks, the body weight of HF females (301±5 g, n = 28) did not significantly exceed that of C dams (291±3, n = 28) before mating, as observed before delivery (467±9, n = 21 and 455±6 g, n = 25, respectively) and just after, since the body weight fall was similar in the two groups (98±4 and 102±5 g, respectively). All lactating HF dams then became gradually thiner and their body weight at weaning was markedly lower than that of control dams (319±7 g, n = 21 versus 351±5 g, n = 25, p<0.0001).This pattern is illustrated in Figure 5 for HF and C dams. The proportion of HF females which become pregnant was smaller than in the control group (23 vs 28 out of 28 females per group) as was the litter size at birth (n = 11.2±0.6 vs n = 13.1±0.7, p<0.05). Regardless of gender, the mean birthweight of pups issued from HF dams was lower than that of pups issued from C dams (6.15±0.04 g, n = 227 vs 6.77±0.07 g, n = 286, p<0,0001) and the male/female ratio was similar in the 2 groups (1.14 and 1.03, respectively).

The maternal diet markedly influenced the weaning body weight of pups, which was dramatically lower in males (53.4±1.8 g, n = 48) and females (50.2±2.0 g, n = 48) born to HF dams compared to those born to control dams (77.4±1.6, n = 41 and 73.0±1.0, n = 42, p<0.0001, respectively). The observations were continued on pups issued from 19 litters from HF dams and 23 from C dams.

#### Adult offspring born to HF or C dams

##### • After 7 weeks feeding the chow diet since weaning

The body weight measured after 7 post-weaning weeks on the chow diet is presented in Table 3 for the 4 groups of adult offspring. The effect of the maternal HF or C diet persisted in the two genders: the growth retardation of pups issued from HF dams, which averaged 32% of body weight at weaning, was partially caught up and represented about 17% in male and 12% in female pups. The absolute body weight gain was identical in the two groups of females regardless the maternal diet, while males born to HF dams growed relatively more slowly than those born to C dams. Taking into account the classical sexual dimorphism in the body growth in the rat species, results were then analyzed separately for each gender (table 3).

**Table 3:** Body weights and post-weaning body weight gains of offspring born to dams fed the chow (C) or the high-fat (H) diet and fed the chow diet for 7 weeks since weaning.

*Gender*	*Male*		*Female*	
Maternal Diet	Chow	High-fat	Chow	High-fat
Group	MC	MH	FC	FH
(n)	(42)	(48)	(40)	(48)
Body weight (g)	410±7^a^	339±6^b^	244±4^a^	214±3^b^
Post-weaning body weight gain (g)	332±7^a^	288±5^b^	171±4^a^	167±3^a^

Different superscript letters ^a,b^ denote significant differences at p<0.05 by ANOVA and the Fisher posthoc test.

##### • Challenged for the highly palatable P diet or maintained on the control C diet

**Figure 6:**
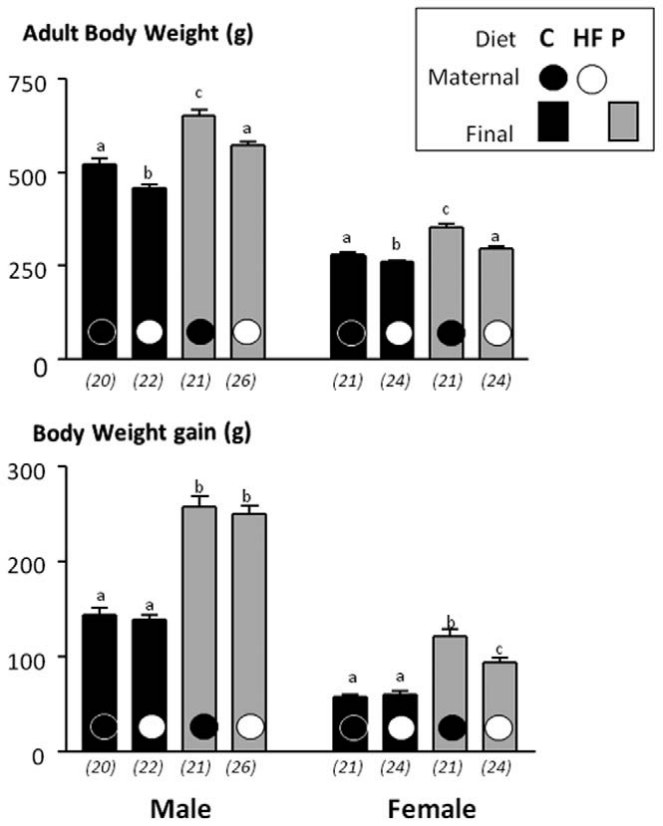
Impact of post weaning diet on the body weight of male and female pups born to dams fed the control diet (C) or high-fat diet (HF). At weaning offspring were divided into four groups of each gender (CC, CH, PC, PH; n from 20 to 26) and named according to the post weaning diet (C or P, first letter) and to maternal diet (C or H, second letter). From weaning to 7 weeks of age all groups were assigned to chow diet then swithched to C or P diet for 13 additional weeks. Body weight and body weight gain are reported in panel A and B, respectively. Different superscript letters ^a,b,c^ denote significant differences at p<0.05 by ANOVA and the Fisher posthoc test.

As shown in Figure 6, swithching from C diet to the P diet for additional 3 months markedly increased the body weight for both genders but regardless the diet, rats born to HF dams still displayed a lower final body weight than their counterparts born to control dams (Fig. 6). The effect of the maternal diet was also observed for the corpulence index (Table 4) which was similar in offspring born to C dams and fed the C diet and those born to HF dams and fed the P diet. The maternal diet did not influence the absolute weight gain (measured between week 7 and the end of the experiment and corresponding to the switch to P diet) of males under the C or P diet (Fig. 6), while females issued from HF dams gained significantly less body weight, under the P diet as compared to those issued from C dams (Fig. 6).

**Table 4:** Corpulence and plasma parameters measured in male and females offspring of dams fed the control or high-fat diet (C or H as 3^rd^ letter) after feeding the control or highly palatable diet (C or P as 2^nd^ letter) for three months.

*Males*	*MCC*	*MCH*	*MPC*	*MPH*
*(n)*	*(20)*	*(22)*	*(21)*	*(26)*
Body weight (g)	524±13^b^	458±11^a^	653±17^d^	573±11^c^
Naso-anal length (cm)	25.67±0.18^b^	24.82±0.16^a^	26.79±0.25^c^	26.27±0.17^c^
Corpulence index*	0.83±0.02^b^	0.77±0.02^a^	0.94±0.02^c^	0.86±0.01^b^
Body weight gain (g)	144±7^a^	139±5^a^	259±11^b^	251±10^b^
Plasma glucose (g/L)	0.93±0.01^a^	0.95±0.01^a^	1.01±0.02^b^	1.00±0.02^b^
Plasma insulin (ng/mL)	0.44±0.04^a^	0.36±0.05^a^	1.24±0.12^b^	1.16±0.10^b^
Plasma leptin (ng/mL)	3.55±0.48^b^	2.98±0.45^a^	12.61±0.84^c^	12.80±1.46^c^
Plasma Triglycerides (g/L)	0.89±0.07^b^	0.73±0.05^a^	1.83±0.13^c^	1.28±0.12^b^
Plasma Cholesterol (g/L)	0.63±0.03	0.55±0.02	0.60±0.04	0.56±0.02

*Females*	*FCC*	*FCH*	*FPC*	*FPH*
*(n)*	*(21)*	*(24)*	*(21)*	*(24)*
Body weight (g)	280±6^ab^	260±6^a^	353±10^c^	295±7^b^
Naso-anal length (cm)	21.2±0.2^a^	21.1±0.2^a^	22.1±0.2^b^	21.3±0.2^a^
Corpulence index*	0.66±0.02^ab^	0.62±0.01^a^	0.75±0.02^c^	0.68±0.01^b^
Body weight gain (g)	58±2^a^	61±3^a^	121±8^c^	94±2^b^
Plasma glucose (g/L)	0.90±0.01	0.95±0.02	0.94±0.01	0.94±0.01
Plasma insulin (ng/mL)	0.23±0.02^a^	0.26±0.02^a^	0.53±0.06^b^	0.53±0.05^b^
Plasma leptin (ng/mL)	1.93±0.22^b^	1.68±0.21^a^	6.47±0.78^d^	5.22±0.52^c^
Plasma Triglycerides (g/L)	0.46±0.02^b^	0.41±0.02^a^	0.78±0.07^d^	0.55±0.03^c^
Plasma Cholesterol (g/L)	0.67±0.03^a^	0.66±0.02^a^	0.76±0.05^b^	0.74±0.03^b^

Different superscript letters ^a,b,c,d^ denote significant differences at p<0.05 by ANOVA and the Fisher posthoc test.

Compared to the C diet, the P diet increased plasma TG, insulin and leptin levels in both genders, and glucose only in males. In both genders and for each diet, plasma TG levels were lower in rats born to HF dams than in those born to C dams. Similar variations were observed for leptin levels, which appeared to be more influenced by the maternal diet than insulin levels. Plasma leptin levels were lower in female born to HF dams and fed P diet compared to those born to C dams and fed P diet. In females but not in males, cholesterol varied in parallel with TG, with higher values under the P diet (Table 4).

Daily food intake was measured during 8 days, one month after the dietary challenge, and the daily energy intake was calculated, as shown in Table 5. Independently of their diet (C or P), the relative daily energy intake is significantly increased in males born to HF dams. In females born to HF dams relative daily energy Intake was reduced when fed control diet, but when fed P diet offspring born to HF or C dams exhibited similar relative energy intake (Table 5).

**Table 5:** Daily energy intake (calculated by animal and by 100 g body weight) in male and female 16 week-old rats (2 or 3 by cage), after 4 weeks feeding the palatable P diet or maintained on the chow C diet since weaning (P or C, respectively as 2^nd^ letter), according to the maternal chow or high-fat diet (C or H, respectively as 3rd letter).

*Male rats*	*MCC*	*MCH*	*MPC*	*MPH*
*(n)*	*(21)*	*(21)*	*(22)*	*(29)*
Body weights (g)	483±12^b^	417±11^a^	546±15^c^	459±9^ab^
Daily energy intake (kcal/rat)	88.5±2.7^a^	86.7±3.1^a^	101.7±2.6^b^	95±2.2^b^
Relative daily energy intake (kcal/100 g body weight)	18.7±0.4^a^	20.9±0.4^b^	17.9±0.7^a^	20.4±0.5^b^

*Female rats*	*FCC*	*FCH*	*FPC*	*FPH*
*(n)*	*(18)*	*(24)*	*(18)*	*(29)*
Body weights (g)	266±6^a^	248±4^a^	317±10^b^	265±5^a^
Daily energy intake (kcal/rat)	66.1±1.6^ab^	60.4±2.0^a^	67.7±2.3^b^	61.4±1.2^b^
Relative daily energy intake (kcal/100 g body weight)	24.7±0.7^b^	24.0±0.5^b^	21.6±1.1^a^	22.9±0.6^ab^

Different superscript letters ^a,b^ denote significant differences at p<0.05 by ANOVA and the Fisher posthoc test.

#### Impact on key genes involved on energy homeostasis

To evaluate the impact of maternal C or HF diet on the hypothalamic and hepatic gene expression levels of male and female offspring challenged with high palatable diet, we have focused our interest on some key genes involved in the control of food intake, energy homeostasis or insulin-sensitivity.

**Figure 7:**
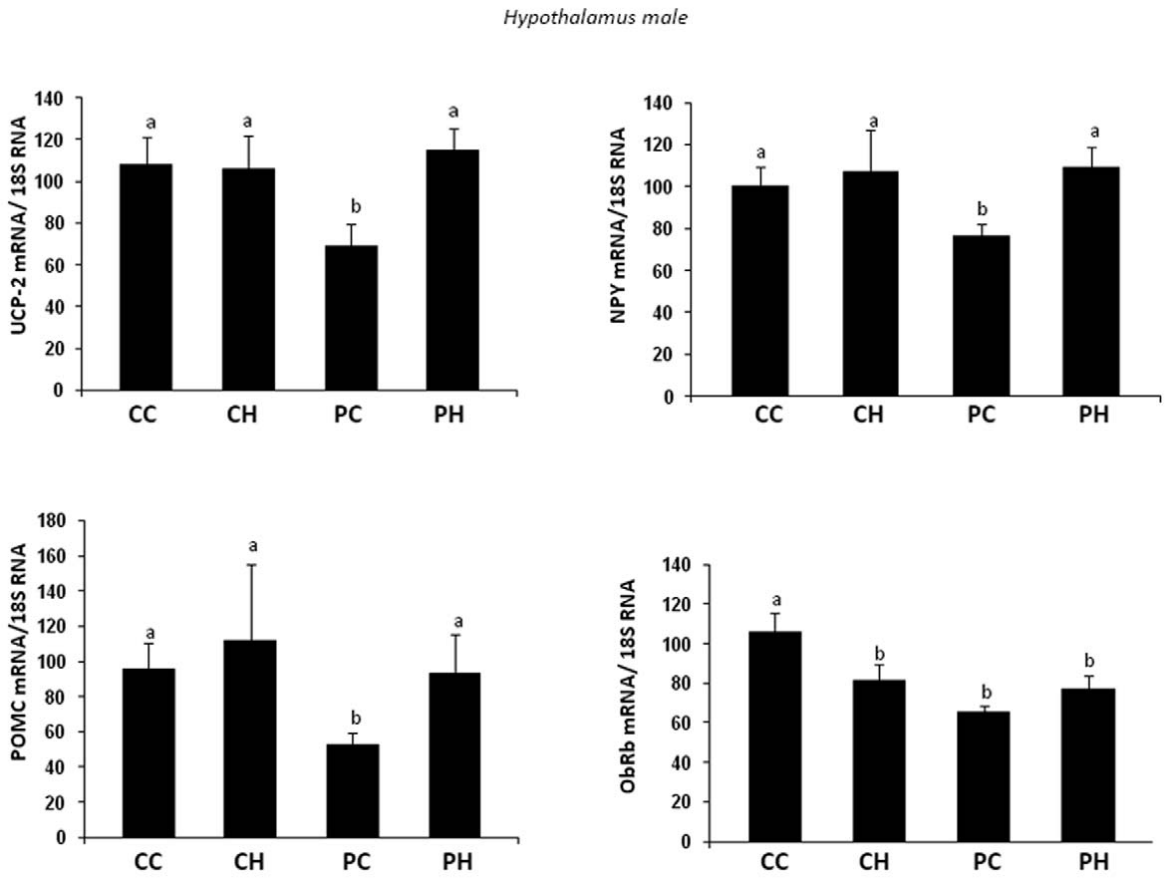
Impact of post weaning diet on hypothalamic gene expression of male pups born to dams fed the control diet (C) or high-fat diet (HF). At weaning offspring were divided into four groups of each gender (CC, CH, PC, PH; n = 10) and named according to the post weaning diet (C or P, first letter) and to maternal diet (C or H, second letter). From weaning to 7 weeks of age all groups were assigned to chow diet then swithched to C or P diet for 13 additional weeks. UCP-2, NPY, POMC and ObRb expression were measured by quantitative RT-PCR and results were normalized to 18S RNA. Different superscript letters ^a,b^ denote significant differences at p<0.05 by ANOVA and the Fisher posthoc test.

**Figure 8:**
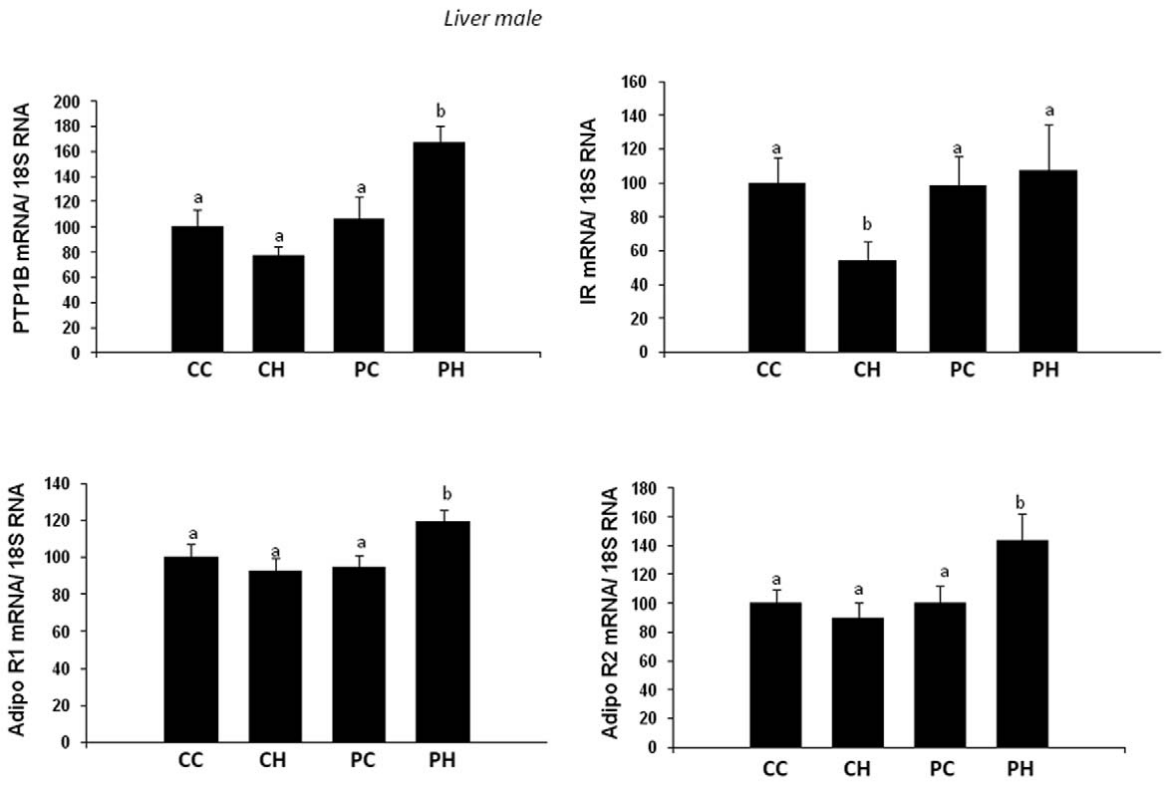
Impact of post weaning diet on hepatic gene expression of male pups born to dams fed the control diet (C) or high-fat diet (HF). At weaning offspring were divided into four groups of each gender (CC, CH, PC, PH; n = 10) and named according to the post weaning diet (C or P, first letter) and to maternal diet (C or H, second letter). From weaning to 7 weeks of age all groups were assigned to chow diet then swithched to C or P diet for 13 additional weeks. UCP-2, NPY, POMC and ObRb expression were measured by quantitative RT-PCR and results were normalized to 18S RNA. Different superscript letters ^a,b^ denote significant differences at p<0.05 by ANOVA and the Fisher posthoc test.

In male offspring, the hypothalamic expression of UCP2, NPY and POMC was lower in PC group than in the other groups (Fig. 7). Moreover ObRb was clearly less expressed in PC and PH groups as compared to CC group, with a similar tendency for CH group (Fig. 7). In contrast, IR expression was not affected by dietary conditions (data not shown). We have also shown that POMC and AgRp expression were not affected by dams' diet by immunohistochemistry (Supporting Data, Fig S1). In the liver, the expression level of phosphotyrosine phosphatase 1B (PTP-1B) was significantly increased in PH group as compared to the other groups (Fig. 8), and a similar effect was found for the expression of adiponectin receptors 1 and 2. Insulin receptor expression was significantly reduced in CH group as compared to the others.

**Figure 9:**
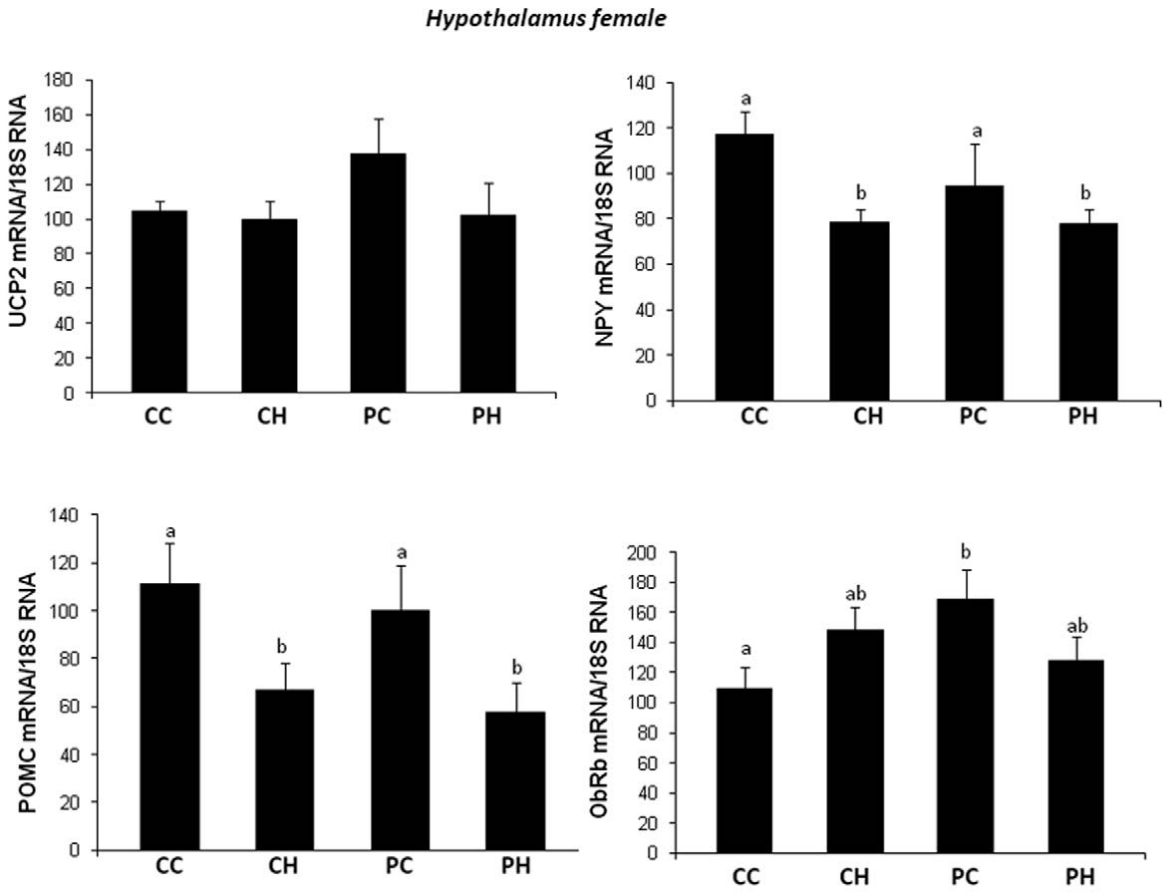
Impact of post weaning diet on hypothalamic gene expression of female pups born to dams fed the control diet (C) or high-fat diet (HF). At weaning offspring were divided into four groups of each gender (CC, CH, PC, PH; n = 10) and named according to the post weaning diet (C or P, first letter) and to maternal diet (C or H, second letter). From weaning to 7 weeks of age all groups were assigned to chow diet then swithched to C or P diet for 13 additional weeks. UCP-2, NPY, POMC and ObRb expression were measured by quantitative RT-PCR and results were normalized to 18S RNA. Different superscript letters ^a,b^ denote significant differences at p<0.05 by ANOVA and the Fisher posthoc test.

In female offspring, hypothalamic expression of POMC and NPY was affected by maternal diet independently of offspring diet (fig. 9) and expression of Obrb was only increased in PC group whereas UCP2 expression was not affected (fig. 9). In liver, no significant change was observed for PTP-1B, IR, AdipoR1 or AdipoR2 (data not shown).

### Impact of a HF diet on the cytoarchitectonic organization of the hypothalamus

**Figure 10:**
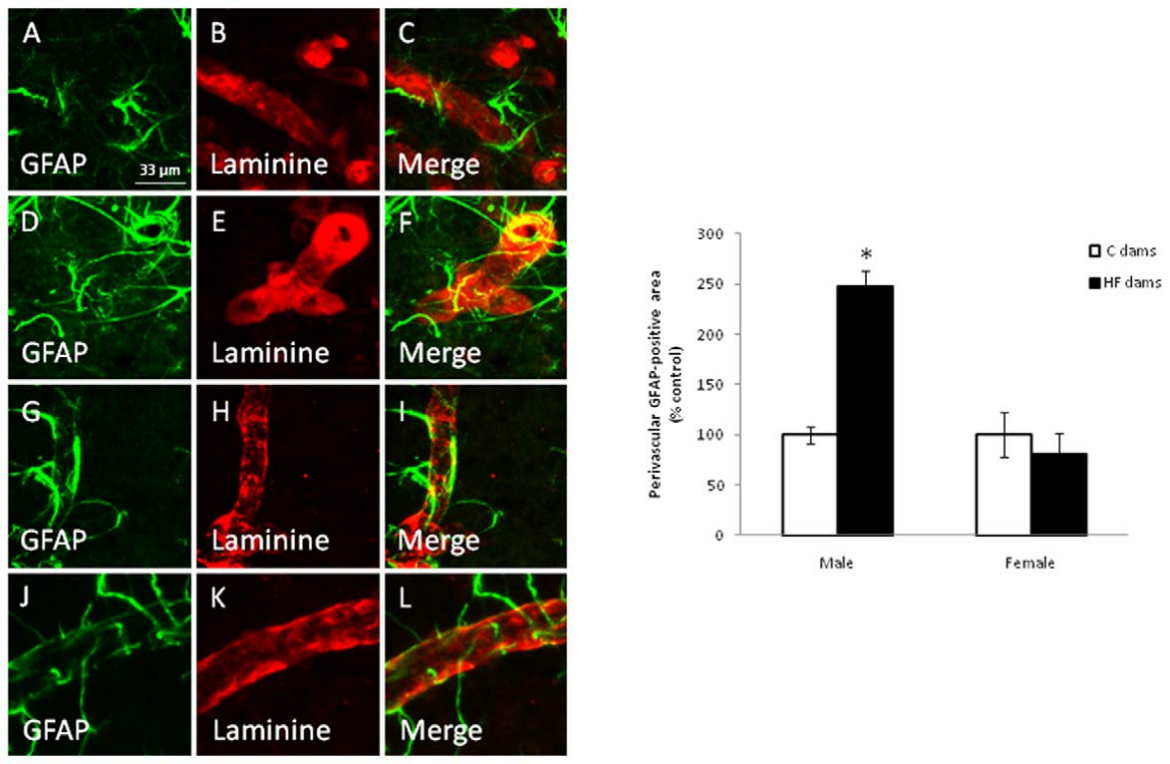
Co-detection of a glial marker (GFAP) and an endothelial marker (laminin) in male (A–F) and female (G–L) offspring born to dams fed a control (A–C ; G–I) or a high-fat (D–F ; J–L) diet in the arcuate nucleus (ARC) at weaning. Maternal HF diet significantly increases the density of astrocytic processes around the blood vessels in males (p<0.05) but not in females (D–F, M). Scale bars = 33 µm.

Since high fat diet given to dams seemed to deeply affect the offspring energy homeostasis, we hypothesized that this could be associated to changes in hypothalamic and more precisely in arcuate nucleus organization. The immunohistochemical detection in the ARC revealed that the maternal HF diet induced a significant increase in the density of astrocytic processes around the blood vessels in males (p<0.05) at weaning whereas this alteration was not observed in females (fig. 10). This gender-specific modification was maintained until adulthood (data not shown). It is to notice that the maternal HF diet had no effect on the vascularisation or the global astrocyte coverage in the ARC, whatever the gender.

## Discussion

The highly palatable P diet used in the present study has been initally presented as an alternative to the classical cafeteria diet to promote a massive obesity [27], [28]. Thus the P diet induced a massive obesity in dams, which was persistent from before mating and throughout gestation and lactation as pups were reared in large litters. At weaning, pups born to P dams exhibited slight growth retardation as compared with those born to control dams. This observation might be surprising since stress is likely minimized in pups weaned on day 28 (instead of day 21), which progressively complete milk by the maternal solid food, as under natural conditions. For comparison with our previous study, dams fed the HF diet (60% energy as palm oil) only presented a slight overweight before mating, followed by a spectacular body weight loss during the lactation period [22] and weaning pups weighed 10% less than those of normally fed dams. Using a HF diet based also on vegetal oil, others reported that gestation/lactation alleviate some of the effect of HF feeding on body weight gain of dams compared to nonpregnant rats but at day 20, pups reared in small litters appeared heavier and fatter, and considered to be more predisposed to obesity [19].

Among the four groups of adult male rats born to C or P dams and weaned on the C or P diet, only the control CC group exhibited an increased phosphorylation level of both STAT3 and ERK1/2 in the hypothalamus in response to leptin challenge. It may be concluded that in the three other groups, a central leptin-resistance was either induced by the post-weaning P diet (PP and PC groups) and/or programmed by the maternal P diet (CP group). Interestingly, only rats fed the post-weaning P diet were overtly obese with classical associated traits of the metabolic syndrome, such as hyperglycemia, hypertriglyceridemia, hyperinsulinemia and hyperleptinemia on fasting state. Those born to P dams and weaned on the balanced C diet (CP group) displayed a normal corpulence and their plasma parameters were quite similar to those of control rats, as reflected by normal body composition. Thus, the defective central leptin signaling, inherited by the offspring of obese dams, is quiescent in these animals which display no tendency to become overweighed even after 5 post-weaning months on the control diet. The physiological significance of this observation is not yet understood. Unexpectedly, the degree of obesity induced by the post-weaning P diet was not exacerbated in offspring born to obese dams and plasma parameters were similar in both groups of leptin-resistant rats, except higher insulin and HOMA values and lower cholesterol level, in the PP than in the PC group. It is to note that the food efficiency of the highly palatable P diet was higher in the PP than in the PC group, suggesting that the maternal P diet programmed a “thrifty” phenotype which tended to minimize the degree of diet-induced obesity in the offspring, as a predictive adaptive response to the obesogenic diet [23]. In the same way, the inherited “spendrift” phenotype observed in offspring born to HF dams, when maintained on the same HF diet, probably accounts for their unexpected resistance to the HF diet [22]. In order to verify whether a maternal HF diet protects offspring from developing obesity and metabolic/endocrine alterations, adult offsprings born to HF or control dams and weaned on a chow diet were submitted thereafter to the obesogenic P diet. In both genders, offspring born to HF dams and fed the C or P diet exhibited lower body weight as compared to their counterparts born to control dam. Thus, the maternal HF diet clearly affects body weight gain of pups, which confirms our previous data [22]. In addition, but only in males, the daily energy intake was higher for PH and CH groups than for PC and CC groups, respectively. This suggests that male offspring of HF dams exhibited higher energy expenditure which may account for their lower body weight and corpulence index. In both gender, the P diet given in adulthood clearly increased the plasma leptin levels which reached the same final value in PC and PH groups, regardless the maternal diet. In females, plasma leptin was lower in PH group than in PC group, likely in relation with the difference in body weight gain between the two groups. Interestingly, in both genders, TG plasma levels were lower in PH than in PC group, reflecting a potential protective effect of the maternal HF diet against adverse effects of the P diet on offspring.

In an attempt to understand mechanisms underlying the potential protective action of maternal high fat diet, we have examined the hypothalamic and hepatic expression of key genes involved in energy homeostasis, and also the astrocyte organization in the hypothalamic ARC nuclei.

In male offspring, the hypothalamic expression level of UCP-2 was significantly reduced in PC group as compared to the other groups and specifically to PH group. Thus, the maternal HF diet contributed to maintain UCP-2 expression level in PH group similar to that of CC and CH groups and this may explain, at least partially, the lower body weight of this group as compared to PC group. It has been reported that mitochondrial respiration in the hypothalamus is dependent upon UCP-2 which is involved in POMC neurons plasticity and also in NPY/AgRP activation in the fasted state [29], [30]. Thus the alteration of UCP-2 expression may affect energy homeostasis in PC group. Furthermore, UCP-2 has been described to protect hypothalamic cells from inflammation damage induced by TNF alpha [31]. This hypothesis is reinforced by the fact that both POMC and NPY expressions were affected in PC group as compared to PH group. The level of ObRb expression was affected in PC and PH as compared to CC group which may be associated to the higher circulating leptin levels. Interestingly, in male offspring liver, PH goup exhibited a higher expression level of Adiponectin receptors R1/R2 as compared to the other groups. AdipoR2 in liver is associated to increased fatty acid β oxidation and reduction of circulating TG [32], this is in good agreement with our data where body weight was lower and relative daily energy intake was higher in PH group as compared to PC group. Furthermore, the TG plasma level is lower in PH group as compared to PC and this could result, at least partially, from the overexpression of liver AdipoR1/R2 in PH group.

These results contrast with those obtained in females, where hypothalamic UCP-2 expression levels were similar in all studied groups whereas maternal HF diet seemed to affect NPY and POMC expression levels in CH and PH groups. In liver, all studied genes were not affected in females (data not shown). This suggests that HF diet given to dams protects male and female offspring, from adverse effects of high palatable diet at least at the level of corpulence index and metabolic markers such as reduced TG, through probably gender-dependent mechanisms. This hypothesis is reinforced by the fact that the maternal HF diet induced a significant increase in the arcuate nucleus density of astrocytic processes around the blood vessels in males but not in females at weaning. This gender-specific modification was maintained until adulthood. It is to notice that the maternal HF diet had no effect on the vascularisation or the global astrocyte coverage in the ARC, whatever the gender. This gender-dependent change in the astrocytic coverage is probably due to sexual dimorphism. Testosterone exposure has been shown to induce significant increase in stellation response in ARC atrocytes [33]. The sexual differentiation of astrocyte morphology has been also reported in other brain areas such as preoptic area where testosterone induced significant modifications in process length and number of astrocytes [34]. Thus, the increased density of astrocytes in male offspring of HF dams may contribute to the formation of synapses and their efficacy leading to establishment of synaptic patterning.

In female offspring of HF dams, the protective effect of maternal HF diet is most likely due to mechanisms that are not yet identified but it is noteworthy to take into account the reduced expression levels of NPY and POMC at the hypothalamic level in PH and CH female groups as compared to PC and CC groups. Since NPY is an orexigenic neuropeptide this may at least partially explain the protective effect of maternal HF diet by limiting then food intake despite the challenge with the P diet leading to reduced body weight gain.

Taken together, our data show that offspring born to overtly obese dams fed a highly palatable P diet beared a defective leptin signaling in hypothalamus, which remained silencious in pups weaned on the chow diet, thus without impact on their predisposition to develop obesity, a situation observed in fructose-fed rats [35] and in our previous study using a HF diet based on palm oil [22].

Interestingly, when offspring born to dams fed P or H diet were compared (experiment 1 and 2), this clearly points out the protective effect of HF diet given to dams. Because the offspring of HF diet dams are less exposed to body weight gain even when fed palatable diet. The protective effect of maternal HF diet involves gender-dependent mechanisms.

## Materials and Methods

### Ethics statement

Rat studies were carried out in agreement with the French legislation on animal experimentation and with the authorization of the French Ministry of Agriculture (Animal Health and Protection Directorate).

### Diets

The commercial chow diet (C, formula 113 from Safe, F-89290 Augy) contained 55.9% starch, 20% protein, 4.5% lipid and was used as ground (experiment 1) or pellets (experiment 2). The semi-solid highly palatable P diet (experiments 1 and 2) was custom-made in our laboratory according to the described formula (Holemans Ket al, 2004) using 33% ground commercial chow (Safe 113), 33% full fat sweetened condensed milk, 7% sucrose and 27% water. The semi-purified HF diet (experiment 2), adapted from Guo and Jen [19], contained palm oil as the main source of fat, as detailed [22]. The energy content and distribution (as carbohydrates, protein and lipids) are given in Table 1 for the 3 experimental diets. Concerning the highly palatable P diet, the accurate measurement of food and energy intake required a conversion factor between the weight of fresh semi-solid preparation and its equivalent weight after deshydratation: the value of 0.66, for the dry/wet diet weight ratio, was obtained experimentally by dehydratation of the P diet under vacuum. For all the diets, and especially the ground chow diet, food intakes were calculated after subtracting the amount of spilled food, estimated by sifting the litters.

### Care and maintenance of animals

Wistar rats were purchased from CER Janvier (Le Genest-St-Isle, France) and maintained under controlled temperature (22±1°C), with a 12–12 h light-dark cycle (light on: 8:00 am) with food and water provided *ad libitum*. The studies were carried out in agreement with the French legislation on animal experimentation and with the authorization of the French Ministry of Agriculture (Animal Health and Protection Directorate).

#### Experiment 1

Thirty-two females and 8 males (aged 8-weeks) were housed by 4 in collective cages and given commercial pellets for one week for adaptation. Two groups of 16 females were then formed according to the C (powdered Safe 113) or P (highly palatable semi-solid preparation) diet given until the end of the lactation period, and males received commercial pellets (Safe 113) (Fig. 1). After 6 weeks, one male was introduced in each cage of females for mating in harem and males were permuted every two days for 14 consecutive days. Timing of delivery, litter size and weight were recorded at birth. Litters were adjusted to 10–11 pups for each dam while maintaining sex ratio as close to 1∶1 as possible. The whole litter weight was checked weekly and the individual body weight of pups was registered at weaning, when aged 28 days. Four groups of 25–26 male pups each were formed and named according to the post-weaning diet (C or P as first letter for control or palatable diet, respectively) and maternal diet (C or P as 2^nd^ letter). Rats were caged by 2 and allowed to free access to food and water. Body weights of dams and pups were measured twice a week. Food intake was monitored during the last 4 weeks before mating for dams, and during 4 weeks in the post-weaning period for pups. After 20 weeks on the C or P diet, adult offspring (age: 6 months) were sacrificed (between 9 and 11 a.m.), either in a postprandial state (4 groups), or after overnight food deprivation and 30 min after intraperitoneal injection of recombinant rat leptin (1 mg/kg) or physiological saline. Blood was collected on heparin (10 IU/mL) and tissues (hypothalamus and liver) were quickly removed. The hypothalamus was immediately frozen into liquid nitrogen and the liver was weighed.

#### Experiment 2

Fifty-six old females and 16 males (aged 8-weeks) were caged by 2 and given commercial pellets (Safe 113) for one week of adaptation. Females were randomized into 2 groups (n = 28) according to the control pellet C diet (Safe 113) or the hypercaloric HF diet provided *ad libitum* for 6 weeks before mating and throughout gestation and lactation. Litters were adjusted to 10–12 pups at birth. Pups were randomized into four groups, according to gender and maternal C or high-fat (H) At weaning when aged 26±1 days, all were assigned to the chow diet for 7 weeks, then in each group, half of the animals were switched to the obesogenic P diet for 13 additional weeks, while the others were maintained on the chow diet. The 8 groups were named according to gender (M or F as 1st letter), final diet (C or P as 2nd letter) and maternal diet (C or H as 3^rd^ letter) (Fig. 1). The body weight evolution of rats was registered every week and daily food and energy intakes assessed during the diet challenge. The animals were sacrificed when aged 6 months, after an overnight food deprivation. By analogy with the body mass index (BMI) in humans, a corpulence index (expressed in g/cm^2^) was calculated from the body weight (g) and the naso-anal length (cm). Blood, liver and hypothalamus were removed as above.

### Biochemical analyses

Recombinant rat leptin was produced as previously described [36]. Phospho-STAT-3 (Tyr705), STAT-3, phospho-ERK and ERK antibodies were purchased from Cell Signaling Technology (Danvers, Massachusetts, USA). Secondary antibodies (from mouse and rabbit) conjugated to peroxidase were purchased from Sigma-Aldrich (Missouri, USA). Other chemicals were generally purchased from Sigma-Aldrich (France).

Plasma glucose, cholesterol and triglyceride levels were measured by enzymatic procedures using commercial kits (Elitech, Salon de Provence, France), by means of an automatic analyzer (Abbott VP, Rungis, France). Insulin and leptin were assayed by radioimmunoassay using commercial diagnostic kits (Linco Research, St. Louis, MO, USA). The homeostatic model assessment for insulin resistance was calculated from insulin and glucose concentrations [37].

### Western blot analysis

Samples were prepared as previously described [38]. Briefly, frozen hypothalami were homogenized in lysis buffer (10 mM Tris-HCl (pH 7.5), 150 mM NaCl, 1 mM EGTA, 1 mM EDTA, 0.5% nonidet-P40, 1% Triton X-100, protease inhibitor cocktail (0.35 mg/ml PMSF, 2 µg/ml leupeptin, 2 µg/ml aprotinin) and phosphatase inhibitor cocktail (10 mM sodium fluoride, 1 mM sodium orthovanadate, 20 mM sodium b-glycerophosphate, 10 mM benzamidine). After lysis in ice for 90 min, insoluble materials were removed by centrifugation (15,000 rpm at 4°C for 45 min) and protein concentrations of the resulting lysates were determined using a protein assay kit (Pierce, Perbio Science, France). Proteins (50 µg) were subjected to SDS-PAGE and transferred onto nitrocellulose membranes. Blots were blocked with 5% non-fat milk and then incubated in the presence of appropriate primary and secondary antibodies. Following nitrocellulose membrane washing, targeted proteins were revealed using enhanced chemiluminescence reagents (ECL, Amersham Life Science, France). The intensity of bands was determined using Molecular Imaging apparatus (Vilber Lourmat, France) and BIO-1D software.

### Quantitative RT-PCR

Total RNA from hypothalamus and liver was extracted using Trizol (Invitrogen, France) according to manufacturer's recommendations. 1 µg of total denatured RNA was reverse transcribed, and the resulting cDNAs were submitted to quantitative PCR. The PCR primer sequences used were as follows, UCP-2 forward: 5′TGGCGGTGGTCGGAGATAC3′, reverse: 5′GGCAAGGGAGGTCGTCTGTC3′; NPY forward 5′ATGCTAGGTAACAAACG3′, reverse 5′ATGTAGTGTCGCAGAG3′; POMC forward: 5′AGGTTAAGGAGCAGTGAC3′, reverse: 5′CGTCTATGGAGGTCTGAAGC3′; LEPRb forward 5′ ACCACATACCTCCTCACACTA 3′, reverse 5′ AGCAGTCCAGCCTACACTCTT 3′; AdipoR1 forward 5′GCTGGCCTTTATGCTGCTCG3′, reverse 5′ TCTAGGCCGTAACGGAATTC3′; AdipoR2 forward 5′ ATAGGGCAGATAGGCTGGTTGA3′, reverse 5′GGATCCGGGCAGCATACA3′; 18S forward 5′TCCCCGAGAAGTTTCAGCACAT3′, reverse 5′CTTCCCATCCTTCACGTCCTTC3′. Real-time PCR was carried out using the Step One apparatus (Applied Biosystems, USA) and the Fast SYBR Green Master Mix (Applied Biosystems, USA). A ratio of specific mRNA/18S amplification was calculated, to correct for any difference in efficiency at RT.

### Immunohistochemistry

One-month old male (n = 10) and female (n = 6) rats born to C or HF dams were used for the immunohistochemical detection of glial fibrillary acidic protein (GFAP) and laminin in the ARC. After deep anesthesia with a ketamin (75 mg/kg) and domitor (0,5 mg/kg) cocktail, animals were perfused with 100 mL of phosphate buffered saline (PBS) 1× pH 7.4, followed by 500 mL of 4% paraformaldehyde in PBS 1X. Brain sections (50-µm thick) were cut with a microtome (HM 650V, Thermo Scientific Microm, Walldorf, Germany) before being incubated with a monoclonal mouse anti-GFAP antibody (1∶1000, Sigma) and a rabbit polyclonal anti-laminin antibody (7∶1000, Sigma) for 12 h at 4°C. Primary antibodies were then visualized with a donkey anti-rabbit IgG coupled to FluoProbes-488 (FP-488; Interchim, Montluçon, France) or a donkey anti-mouse coupled to cyanine-5 (Cy5; Jackson Immunoresearch Laboratories; Suffolk, UK) antibodies (1∶500). Immunofluorescence (IF) was examined under a confocal microscope (Zeiss LSM 510 system, Germany). Optical sections were taken through the Z axis at 1 µm intervals and averaged four times. Quantification was performed with ImageJ 1.36b software (NIH, USA). Perivascular GFAP coverage was assessed by measuring the GFAP-positive fraction on blood vessels contours in whole bilateral ARC and after background subtraction. This operation was performed on six different vessels throughout six different sections homogenously distributed through the ARC in each animal.

### Statistical analysis

Statistical analysis was performed using (Stat View Software, ver.5) to detect significant intergroup differences. Values were expressed as means ± SE, and *P<0.05* was considered statistically significant.

## Supporting Information

Figure S1 Detection of α MSH (upper panel) and AgRp (lower panel) in male offspring rats born to dams fed a control (CC) or high-fat diet (HF) in the arcuate nucleus at weaning.
(TIF)
