# Supplementary figures and images for: Unexpected Long-Term Protection of Adult Offspring Born to High-Fat Fed Dams against Obesity Induced by a Sucrose-Rich Diet
Source: PLoS One. 2011 Mar 25;6(3):e18043. doi: 10.1371/journal.pone.0018043 (PMC3064582; doi:10.1371/journal.pone.0018043)

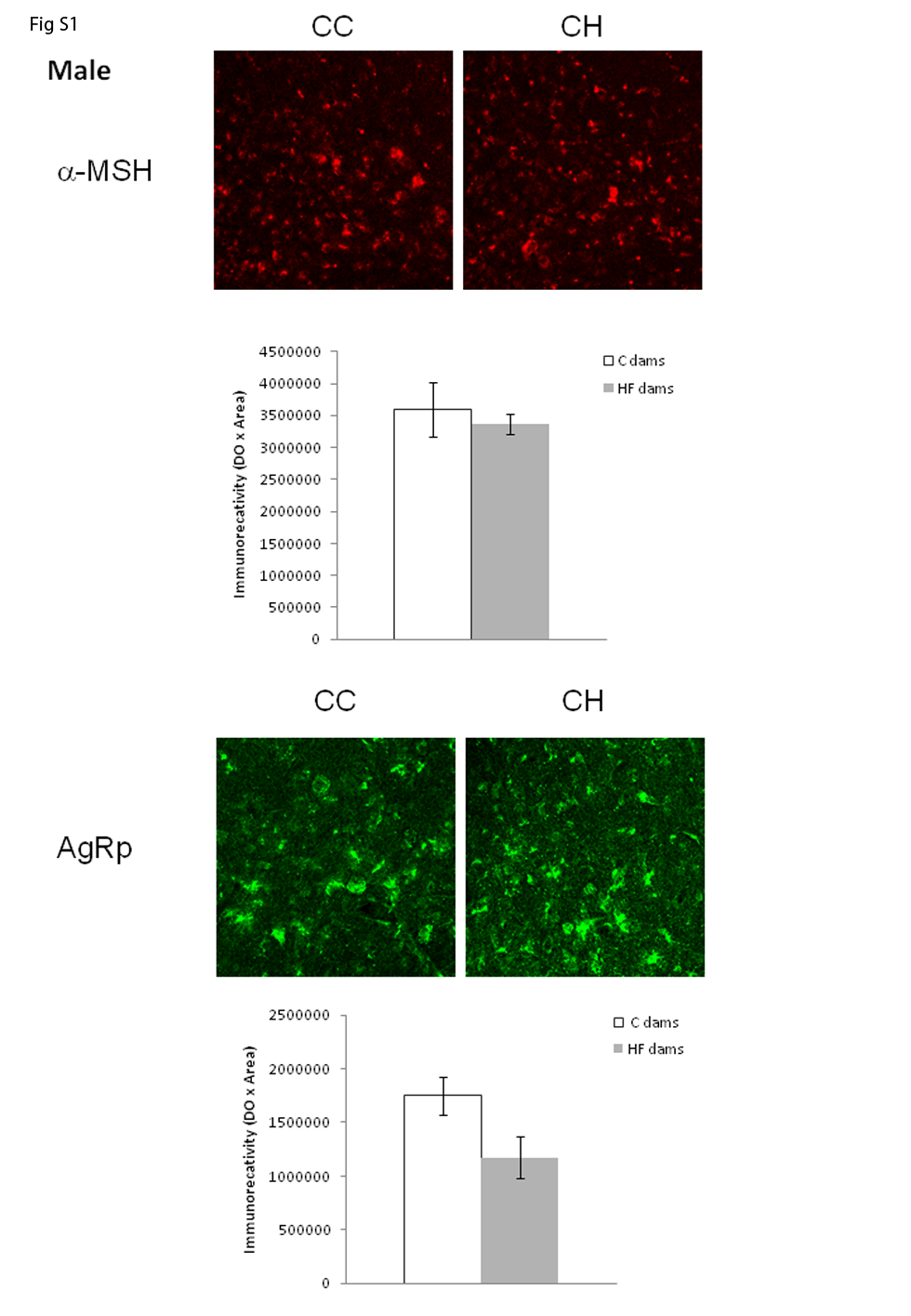

Supplement: Figure S1 — Detection of α MSH (upper panel) and AgRp (lower panel) in male offspring rats born to dams fed a control (CC) or high-fat diet (HF) in the arcuate nucleus at weaning. (TIF) [file pone.0018043.s001.tif]
